# Influence of Drought and Salt Stress on Durum Wheat Grain Quality and Composition: A Review

**DOI:** 10.3390/plants10122599

**Published:** 2021-11-26

**Authors:** Michele Andrea De Santis, Mario Soccio, Maura Nicoletta Laus, Zina Flagella

**Affiliations:** Department of Agriculture, Food, Natural Resources and Engineering (DAFNE), University of Foggia, 71122 Foggia, Italy; mario.soccio@unifg.it (M.S.); maura.laus@unifg.it (M.N.L.)

**Keywords:** durum wheat, drought, water stress, salt stress, gluten proteins, dietary fiber, bioactive compounds, micronutrients, wheat quality

## Abstract

Durum wheat is a staple crop for the Mediterranean diet because of its adaptability to environmental pressure and for its large use in cereal-based food products, such as pasta and bread, as a source of calories and proteins. Durum wheat whole grains are also highly valued for their peculiar amount of dietary fiber and minerals, as well as bioactive compounds of particular interest for their putative health-beneficial properties, including polyphenols, carotenoids, tocopherols, tocotrienols, and phytosterols. In Mediterranean environments, durum wheat is mostly grown under rainfed conditions, where the crop often experiences environmental stresses, especially water deficit and soil salinity that may induce a hyperosmotic stress. In particular, changes in C and N accumulation due to these abiotic conditions, during grain filling, can influence starch and storage protein amount and composition in durum wheat caryopsis, thus influencing yield and quality traits. Recent advancements regarding the influence of water deficit and salinity stress on durum wheat are critically discussed. In particular, a focus on stress-induced changes in (a) grain protein content and composition in relation to technological and health quality; (b) starch and dietary fiber accumulation and composition; (c) phytochemical composition; (d) health-related grain micronutrient accumulation, such as Fe and Zn.

## 1. Introduction

### Crop Relevance, Geographic Distribution and Main Quality Traits

Durum wheat (*Triticum turgidum* L. ssp. *durum*) is a tetraploid (AABB) cereal species mainly cultivated in North America, Mediterranean basin, and Australia. Worldwide production is generally comprised in the range of 35–40 million tons per year. The EU is the top producer in the world, whereas Italy is the leading European country in durum wheat production, followed by France and Turkey (International Grains Council, https://www.igc.int/en/default.aspx, accessed on 10 May 2021). Gluten obtained from durum wheat is generally strong, characterized by high tenacity and low extensibility, typical of semolina flour mainly used for the production of pasta and, in the Mediterranean area, for bread and couscous [1]. Durum wheat is one of the main sources of carbohydrates and proteins, in the Mediterranean diet, also containing dietary fiber and many bioactive components, which are beneficial for human health. The chemical composition of durum wheat kernel can influence both the technological and nutritional properties of the processed products. External pericarp (bran) mainly contains cellulose, minerals, vitamins, and fiber while germ represents 2–3% of seed; indeed, the main fraction is represented by endosperm, which is divided into aleuronic layer (monolayer of cells lost during milling) and amyloid endosperm which represents about 87–89% of the seed. Inside the latter there are mainly starch granules and storage proteins while in the aleuronic layer, proteins, fats, minerals, vitamins, sugars are contained [2].

Proteins are distributed in different percentages depending on the region of the grain, generally ranging from 8% to 20% of the entire grain. Wheat proteins have been classified according to their solubility in albumins (monomeric, soluble in water), globulins (monomeric, soluble in salt solution), gliadins (monomeric, soluble in alcohol), glutenins (soluble in dilute acid or alkali). Albumins and globulins have a structural/metabolic role; together gliadins and glutenins represent about 80% of total proteins in flour and they are the most important determinants of the functional properties of wheat flour. Dough properties depend on the balance between gliadin (contributing to dough viscosity) and glutenin (contributing to the strength and elasticity of dough). Gluten proteins are also known as prolamins for their high contents in proline and glutamine which together account for 30–50% of the total amino acids. Comparison of the amino acid sequences of individual prolamins has led to a classification based on primary-structure relationships. The three groups are: (a) high-molecular-weight (HMW) prolamins: the HMW glutenin sub-units (HMW-GS); (b) sulfur-rich prolamins: the low-molecular-weight glutenin sub-units (LMWGS), α-gliadins, β-gliadins, and γ-gliadins; (c) sulfur-poor prolamins: ω-gliadins [3]. HMW-GS composition and LMW-GS (B-type) have a key role in determining technological performances on dough, especially in terms of tenacity for pasta-making [4].

Carbohydrates represent most of the dry matter in durum wheat grain [5], in the form of starch (55–75%), mono-, di-, and oligosaccharides (4%) and non-starch polysaccharides (10%). Starch is composed of two polymers: linear amylose, in which glucose units are bound together by α (1→4) glycosidic bonds and branched amylopectin which includes linear amilose-like residues arranged to form branches (1→6 α-linkages) each 24–30 glucose units. The proportions of amylose and amylopectin (18–35%) in starch have a significant impact on processing quality, with high amylose starches being preferred for developing healthy foods as they are more slowly digested in the human gastro-intestinal tract and become resistant on cooking, leading to reduced glycemic index [2]. TILLING (targeting induced local lesions in genomes) technique has been recently adopted for manipulation of starch composition in durum wheat for high amylose starch composition [6]. Non-starch polysaccharides (NSP) in durum wheat represent the major part of dietary fiber (DF), mostly arabino-xylans (AX) and mixed-linkage β-glucan (MLG). AX polymers represent about 70% of total wheat grain cell wall NSPs, and consist of a xylose (β-D-xylopyranosyl) backbone (1→4 glycosidic linkages) which may be substituted with arabinose (α-L-arabinopyranosyl) at either the 3 or the 2 and 3 positions. AX is present as water-extractable (WE-AX) and water unextractable (WU-AX) fractions, with average contents of about 0.5 and 1.7 g/100 g flour, respectively [5,7]. MLG are glucose units linked (1→4)-β (as in cellulose) but interspersed with (1→3)-β-linkages. Both WE-AX and MLG contribute to the valuable role of soluble DF in diet, especially for whole meal pasta and baked products. DF content and composition tend to change, moving from the outer layers to the starchy endosperm, with a general reduction of AX and MLG and an increase of AX substitution (branching), leading to a higher solubility of the flours [2]. The role of dietary fiber, and in particular β-glucan, after long term consumption has particular relevance for health by reduction of caloric intake, post-prandial glycemia, prebiotic effect, and post-ischemic cardioprotection [5,8]. Lipids generally consist on 2-3% of grain chemical com-position and they are mainly present in germ; technological relevance of lipids on flourand dough functionality is due to the ability of stabilizing gas cells in breadmaking by theinteraction with gluten proteins and starch [9]

Durum wheat grains also contain numerous bioactive compounds from secondary metabolism, including flavonoids, phenolic acids, alkylresorcinols (ARs), phytosteroids, tocols, carotenoids, and other minor components. Flavonoids are generally concentrated in the germ and the bran of the cereal grains. For durum wheat, a genetic variability in flavonoid and phenols content exists [10], as well as a relationship with the antioxidant activity [11]. Typical phenolic acids found in wheat grain include ferulic acid, p-coumaric acid, syringic acid, vanillic acid, and caffeic acid. These phenolic acids are generally present as soluble-free, soluble-conjugated, and insoluble-bound forms in wheat grain, and most phenolic acids are in the insoluble-bound form. Ferulic acid is the most abundant in durum wheat [12], mostly esterified to arabinose residues on the O-5 position, with the possible formation of cross-links by oxidation of ferulate present on adjacent AX chains [5]. Wheat grain generally contains high levels of alkylresorcinols (ARs), with the length of a saturated alkyl tail between 17 and 25 carbons among which C21:0 accounts for the largest proportion of AR homologues. Specifically, ARs are concentrated in the bran fraction of wheat grain, including the hyaline layer, testa, and inner pericarp, and they are therefore mostly absent in the refined wheat flour and can be used as “biomarkers” for the whole-wheat food consumption in humans [13]. Cereals are considered to be a moderate source of tocols, providing 6–20 mg of α-tocopherol equivalent per gram [13]; wheat germ mainly consists of α- and β-tocopherols. Tocotrienols are concentrated in the pericarp, testa, and the aleurone. In durum wheat, lutein results the main component of carotenoids, followed by zeaxanthin and β-carotene [14]. An increasing interest is also related to dietary lignans for their potential beneficial properties, i.e., anticancerogenic, antioxidant, estrogenic, and antiestrogenic activities [15].

Durum wheat grain is a source of micronutrients; major forms are vitamin E, several B vitamins, and various minerals [16]. These are unevenly distributed in the kernel and are mainly found in wheat germ and the bran; hence, they are found in reduced amounts in refined flours. They play a fundamental role in human health, thus their deficiency affects physical and mental disorders [17]. Micronutrients have also a crucial role in plant nutrition, as they are structurally involved in enzyme activity and metabolism. A deficiency can also influence plant susceptibility to abiotic stresses [18]. The principal investigated health-promoting micronutrients in durum wheat are iron, zinc, manganese, and selenium. A known genetic variation exists, also in relation to environmental conditions [19]. Agronomic management, including nitrogen nutrition [20] or direct biofortification treatments can improve durum wheat nutraceutical properties [21,22,23].

Although a lot of research has been conducted to find out the effects of drought and salinity on durum wheat physiological and biochemical processes and on specific agronomic and qualitative yield traits, less information exists on the effect of these environmental stresses on yield quality considered as a whole in its different aspects. 

Thus, the current review emphasizes the drought and salinity induced changes in grain quality with an integrated view of the traits related to technological and health quality taking also into account the updated studies with new –omics methodologies.

Furthermore, a particular attention to the interactions between genotype and environment on durum wheat quality in a climate change scenario is paid.

## 2. The Influence of Environmental Conditions on Durum Wheat

### 2.1. Climate Change and Environmental Variability

The Mediterranean climate, beyond the Mediterranean basin, is characteristic of those areas of the temperate belt closest to the regions of the great warm deserts of the subtropical and tropical latitudes such as Southern Australia, the Cape Province in South Africa, Central Chile, and Central-Northern California. In this climate zone, unlike the tropical one, the four seasons can be distinguished. The Mediterranean region is characterized by an extremely variable climate, with hot, dry summers and cool, wet winters [24]. In the last decade, increased climatic variability including severe drought events have become a major problem leading to significant yield losses. Indeed, changes in weather and climate observed in the latest century have an anthropogenic origin [25]. Global warming is also associated to an increase in frequency and magnitude of extreme events [26], with drier and warmer trends in Mediterranean countries [27]. The effect of the increased atmospheric CO_2_ levels can be relevant for grain productivity and quality [28,29,30]. Indeed, climate change can potentially have very different impacts on crop performance depending on the time-point and duration of drought events and on the occurrence of drought stress alone or in combination with heat stress [31]. Drought can be described as a condition of deficit between evapotranspiration demand and amount of soil water availability. This is not only the result of an extreme weather event, but can also be determined by the combination of heat and high radiation, limiting the root water uptake and canopy transpiration [32]. In Mediterranean environments, water stress tends to increase throughout grain maturation stages [33], resulting as the major environmental factor affecting durum wheat grain quality, associated to high temperatures [34]. The typical low precipitation of Mediterranean area, especially within sub-coastal regions, can contribute for soil mineral accumulation and salinization, also for crop cultivated under rainfed conditions or in rotation with salt water irrigation such as durum wheat [35].

### 2.2. Effects of Hyperosmotic Stress on Agronomic and Crop Physiological Parameters

Durum wheat is generally cultivated in water-limiting environments, because of the assumption of higher stress tolerance than bread wheat, despite a lower yield potential. On the other hand, attainable and actual yields are the results of water-limiting and abiotic/biotic stress conditions that influence final production, and then quality [36]. In terms of yield components, final grain number (per square meter and per spike) results as one of the most important yield components in determining final yield, especially under water stress conditions [37] also in interaction with nitrogen availability [32]. Hyperosmotic conditions can arise at different growth stages, affecting developmental processes at morphological, physiological, and molecular level with a consequent reduction of final grain yield [38,39]. As a crop mostly grown under rainfed conditions, the exposure to meteorological conditions is critical for agronomic performance. The monitoring of rainfall deficit, i.e., difference between rainfall and evapotranspiration (ET_0_) or even its ratio (aridity index), is strategic for smart management, especially under Mediterranean environment; however, the uncertainty of water use and supply remains a critical aspect that could be managed by crop modeling, in a climate change scenario [40]. Soil physical characteristics significantly affect water holding capacity and water supply, influencing dry matter and mineral accumulation [41]. As for salinity stress, durum wheat is conventionally classified as tolerant herbaceous crops (5.9 dS/m threshold) according to the FAO classification (www.fao.org, accessed on 1 September 2021).

Crop physiological status can be assessed by several destructive and non-destructive techniques, including spectroscopy, thermography, fluorescence also as remote sensing [42]. Spectroscopy in the visible (VIS, 400–700 nm) and near infrared (NIR, 700–2500 nm) electromagnetic spectrum is developed on the basis of plant and crop spectral signature, considering the ability of plant pigments (chlorophyll, carotenoids, anthocyanins) to absorb radiation and to reflect light with increased wavelength (red shift). A large number of spectral reflectance indexes, or vegetation indexes (VI), have been proposed in order to assess morphological, physiological, and biochemical parameters also related to stress. Normalized Difference Vegetation Index (NDVI) is the most widely used VI, originally developed to estimate green biomass and then crop yield [37,43,44]. Other VIs are available in order to assess crop physiological status, also in relation to abiotic stresses, including Photochemical Reflectance Index (PRI), Plant Senescence Reflectance Index (PSRI), SWSI (Salinity and Water Stress Index), WI (Water Index), as reported by Galieni et al. [42]. The assessment of crop physiological status can be assessed at different scales (plant, canopy, farm, region) by proximal and/or satellite remote sensors in precision farming [45] in order to evaluate possible hyperosmotic stresses (water and/or salt) during key growth stages. Indeed, reproductive stages, in particular grain filling, are quite sensitive to abiotic stresses and the effect on quality may be relevant [39,46]. An important genetic variability in terms abiotic stress tolerance exists for durum wheat. The recent findings in candidate genes involved for abiotic stress and quality [47] might be relevant on developing new varieties with specific ideotypes developed under different agronomic and water scenarios, typical of Mediterranean conditions [48].

## 3. Effect of Water Deficit and Salinity Stress on Durum Wheat Grain Quality Traits

Grain development is a critical growth stage for durum wheat quality and involves transport and mobilization processes required for importing various constituents and many biochemical processes for the synthesis of macromolecules: proteins, carbohydrates, nutrients, and secondary metabolism compounds accumulation in the developing seeds. During this stage, drought stress can cause membrane, chlorophyll, and photosynthesis damage due to stomatal or non-stomatal associated mechanisms [49]. Water stress can often occur under anthesis, with a strong impact on yield and quality, influencing grain-filling duration, causing elevated endogenous ABA levels, carbohydrate deprivation, and a reduced ability of reproductive sinks to use starch and sucrose. The reduced starch accumulation under drought stress and the increase in non-reducing sugars may result in ovary abortion, leading to poor grain set and yield [50]. These conditions generally led to a reduction in grain weight and test (specific) weight; the reduction in test weight is also accompanied to higher concentration of nutrients (macro and micro, except for starch) in grains; for this reason test weight is generally recognized as an indicator of stress in grain [50,51,52].Environmental conditions, such as water deficit and salt stress, can affect grain quality and composition [53]; while many studies have been carried out on the effect of water deficit on durum wheat quality, few ones are available on the effect of salinity, which was more investigated on bread wheat. In particular, contrasting results are reported for the different quality traits (protein, fibre, fat, ash, minerals) in relation to salt stress levels and genotype salt-tolerance [54]. 

In the following sections some findings published in the last two decades were reported. The main scientific databases were consulted, in relation to the following keywords: durum wheat, abiotic stress (water deficit/drought, hyperosmotic/salinity/salt stress) and the main chemical compounds involved in technological and health quality (proteins, starch, dietary fibre, micronutrients, bioactive compounds).

**Figure 1:**
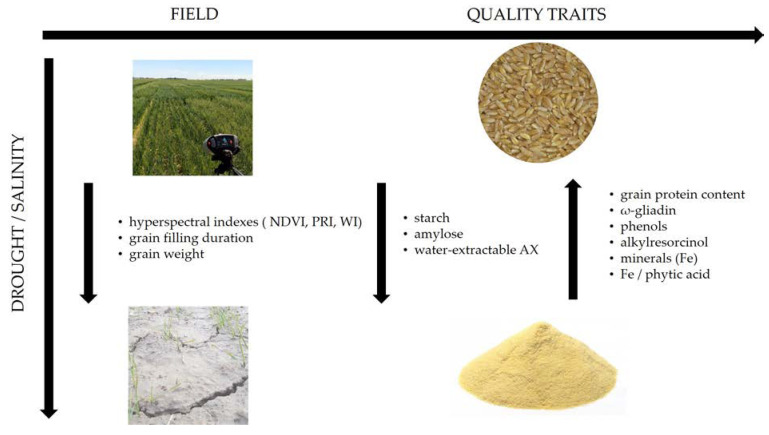
Synthetic representation of the main effects of hyperosmotic stress (drought and salinity) on durum wheat crop physiological and quality traits [7,34,55,56,57,58,59,60,61,62,63].

The main effects on durum wheat quality traits are reported below and, also, summarized in Table 1 and Figure 1.

### 3.1. Storage Proteins

Protein concentration is strongly influenced by hyperosmotic stress in interaction with nitrogen supply (from soil and fertilization) and has a negative relationship with grain yield. The biosynthesis of each protein fraction is not synchronous and the genes regulating their accumulation are distinct from each other [94]; for this reason, the timing of a stress event, such as drought, can influence grain filling duration and then final protein composition [52,95].

The influence of hyperosmotic stress, and in particular water deficit, has been investigated in scientific literature in order to assess the impact on final storage protein composition and, in some cases, on technological quality for durum wheat. Most of the studies are conducted, both in field or controlled conditions (growth chambers, greenhouses) with supplemental water supply by irrigation, in order to assess differences in water use. Other studies reported differences observed in rainfed field conditions, with different rainfall amount and distribution across experimental crop seasons (Table 1).

In most of the studies, a lower water availability (due to rainfall and/or irrigation supply) was associated to a higher grain protein concentration (GPC) in durum wheat grains. The increase was often related to the level of water deficit imposed in the experimental conditions. The effects on gluten-forming proteins is somewhat contrasting.

In Mediterranean environments, a mean increase in protein content, in HMW-GS and in HMW/LMW glutenin subunits ratio was reported under water deficit [34] on two durum wheat genotypes (Ofanto, Simeto) grown in field under rainfed and irrigated conditions. When rainfall deficit occurred during grain filling, also a higher aggregation level of glutenin subunits (UPP) was observed that determined an improvement of technological quality (gluten index).

In recent experiments conducted at Cimmyt (Obregon, Mex), on a larger set of genotypes, the effect of water deficit was investigated in relation to protein content, gluten composition, and technological performances [1,55,65,96]. The experiments were carried out in an arid environment, with different levels of water supply by drip irrigation (moderate and severe drought compared to optimal conditions). Drought stress generally led to an increase in GPC and ω-gliadins fractions on increasing drought intensity, associated to a decrease in HMW-GS concentration. The authors reported no significant mean changes for LMW-GS, γ-gliadins, and α-gliadins [55]. In another study, including 46 durum wheat genotypes from different countries of origins and under comparable experimental conditions, the authors observed that severe drought stress was associated to a slight increase of the alveographic dough strength (AlvW) and extensibility (AlvL) [96].

A field study conducted in Spanish environments [64] dealt with the effect of water deficit under different water supply (irrigated, rainfed moderate dry, and rainfed very dry, i.e., 461, 330, and 180 mm respectively) on a set of 10 durum wheat genotypes (including an advanced selection from Cimmyt). In particular, the authors evaluated the impact of drought on protein content and amino acid (aa) composition and their relationships with grain filling duration (GFD). The imposed water deficit reduced GFD with consequent lower grain yield and higher GPC; most of the genotypes showed a progressive higher aa concentration on increasing stress level, except for glutamine (Glu), phenylalanine (Phe), and proline (Pro). A linear relationship between aa composition and N content in grain was also proposed. This study represented an important milestone in order to describe the changes that occur in aa and protein composition in durum wheat grains.

An evaluation by a proteomic approach (2DE x MS/MS) of the effect of water deficit after anthesis was conducted on two durum wheat genotypes (Ciccio, Svevo) under controlled conditions [56,66]. Under water deficit an increase in gliadins and gliadin-like proteins (such as C- and D-subunits of LMW-GS) and a reduction in typical LMW-GS were observed. The authors reported also the upregulation of several α-gliadins associated with immunological potential under water deficit.

A larger number of papers is present in literature that describe the changes in durum wheat storage proteins due to crop seasonal variability, in particular associated to differences in rainfall amount and distribution in the same environment. In a study conducted under conservative agriculture in Central/South Italy [20,67], the accumulation pattern of gliadins and glutenins sub-fractions of cv. Iride was investigated in relation to N fertilization rate and source (urea and calcium nitrate) in two crop seasons characterized by a different rainfall distribution (2011 wetter, 2012 dryer). Beside the higher protein content due to the higher N supply, the authors observed a −30% accumulation in glutenins (both HMW-GS and LMW-GS) in the dryer crop season, with a consequent reduction of the GS/glia (−40% with urea, −47% with calcium nitrate). The authors attributed these differences also to a late water stress occurring in 2012, also described by hyperspectral crop phenotyping. The same group investigated, on a different cv. (Saragolla), the interaction with tillage and cropping systems [68]. The authors reported a significant interaction between crop seasons (2017 markedly dryer than 2016) in terms of protein composition, with a higher gliadin accumulation in the dryer one and a significant interaction with management in relation to HMW-GS/LMW-GS [68].

Another study by Ferrise et al. [75,76] described the changes in GPC and protein composition in relation to sowing date and N fertilization rate by the use of a crop model (SiriusQuality1) on the basis of field trials conducted in central Italy (University of Florence) on cv. Creso under two contrasting crop seasons in terms of rainfall amount, especially during reproductive stage. Crop season differences were related also to sowing dates, since longer vegetative period resulted in higher accumulation (dry matter, N) and then higher grain yield, with consequent dilution of grain N, also related to N supply. The authors also stated that efficiency of DM and N remobilization in durum wheat increases slightly with N availability and decreases in response to post-anthesis water deficit and the insolubilization of glutenin polymers is not directly related to the rapid loss of water after physiological maturity, but rather to the continuous dehydration of the grain.

Another set of experiments, conducted in Sardinia [73,74], assessed the environmental effects mainly due to differences in seasonal rainfall amount and distribution. These studies included durum wheat genotypes with different release dates; the authors reported minor mean changes in terms of protein content between wetter and dryer crop seasons, and also contrasting results in terms of gliadin/glutenin ratio, possibly explained by the large genetic variability [73]. The authors also reported that, in the dryer crop seasons, monomeric gliadins and the S-poor/S-rich ratio are the main fractions affected [74]. Additionally, a set of studies conducted in South Italy described the phenotypic differences of durum wheat genotypes, with different release dates (including old Senatore Cappelli and modern Saragolla), under rainfed crop seasons characterized by differences in rainfall distribution in the final grain filling stages [57,58,69,70,71]. As an outcome from these studies, the dryer crop seasons generally led to a higher GPC and a higher ω-gliadin (in particular ω5- or Tri a 19 allergen), assessed also by immunological and proteomic approach [57,70]. The effects on HMW-GS and gliadin/glutenin resulted somewhat contrasting and related to the genetic variability, with a positive relationship between S-poor fractions (HMW-GS, ω-gliadins) and S-rich fractions (LMW-GS, γ-gliadins) with GPC [57,72]. As for the accumulation of proteins containing peptides involved in celiac disease (immunological and toxic, G12/33-mer) a trend of a negative relationship between their prevalence in grains and the rainfall amount during grain filling period was observed [58,70,71,72]. These implications might be of high relevance for the consumers.

Fewer studies are available in relation to the effect of salinity stress on grain protein composition in durum wheat [35,77,78]. Two independent investigations conducted in greenhouse, with three salinity stress levels (control, moderate stress, and severe stress), showed contrasting results; the study conducted by Katerji et al. [77] showed no difference in GPC with different salinity levels (in two varieties with different salt-tolerance), while Borrelli et al. [35] reported an increasing trend of GPC with salt stress levels, with a significant improvement of technological performance, suggesting positive implications in terms of gluten-forming proteins expression. Indeed, the authors of [78] reported a higher gluten content in the salt-stressed thesis than in the relative controls, in accordance with Houshmand et al. [97], who found an improvement of technological performance (SDS sedimentation). A study available in literature on the effect of salt stress on gluten protein composition was conducted on bread wheat [79], where the authors observed a positive relationship between salt stress (6 levels) and the expression of HMW-GS.

### 3.2. Starch and Non-Starch Polysaccharides

The dynamics of carbohydrate accumulation in grain is well documented in cereals, including durum wheat [98], and how water limitation during grain filling reduces final grain weight and, in particular, starch accumulation [52]. Water limitation in wheat reduces amylose content and starch granules (A-, B-, C-) size in endosperm [99,100]. On the other hand, Graziano et al. [88] observed an increase in A-type granule starch in the crop seasons with less rainfall. Durum wheat non-starch polysaccharides mainly consist of arabinoxylan and (1–3) (1–4)-β-d-glucan (β-glucan), which represent the major components of dietary fiber [5]. Genetic and environmental factors can influence the quantity and composition of DF [84,85]. Unfortunately, most of the studies are focused on bread wheat. Contrasting outcomes are available in relation to drought after flowering for AX content. In [101], the authors observed a positive relationship between drought and total AX in grains, which negatively correlated with starch content. The changes in terms of AX composition are strictly related to the degree of substitution of the AX with arabinose (termed branching). In another study it was observed that AX accumulation proceed from a highly to a less branched (arabinosylation) form [102]; for this reason, the timing of drought stress may impact on A/X ratio and AX solubility. Rakszegi et al. [59] evaluated the effect of drought and heat (single and combined) on three wheat genotypes under controlled conditions, observing a GxE interaction, explained by different drought tolerance mechanisms [86]. AX generally increased under drought, with a reduction of arabinosylation (% unsubstituted AX oligo-saccharides, AXOS) and a consequent lower WE-AX content in only one genotype (Mv-Magma). MLG content resulted negatively affected by drought, with no marked changes in terms of gluco-oligosaccharide composition (with 3 or 4 glucose residues, DP3:DP4 or G3:G4), assessed by enzyme fingerprint. Indeed, in a set of experiments conducted in field within the EU funded HEALTHGRAIN project, it was observed a positive correlation between rainfall amount, in particular during grain filling, and WE-AX [84,85], indicating how soil water availability can influence AX solubility. The same trend was confirmed in a subsequent experiment, under greenhouse conditions, with lower tot-AX and WE-AX with drought and a positive correlation between WE-AX and mono-substituted AXOS, in some lines [86].

As for durum wheat, Lempereur et al. [80] explored the changes in AX in five genotypes grown in two environments, in a range of water (rainfed and sprinkler irrigation) and N supply (52, 102 kg/ha), showing a higher tot-AX content under low water availability. Under Mediterranean conditions, the study conducted by [81] reported the tot-AX and WE-AX content of a set of 10 genotypes, grown in different environments with different rainfall amounts during vegetative cycle, showing a significant GxE interaction. A further investigation on a set of genotypes, grouped into old and modern ones, was conducted in rainfed conditions in south Italy [7,82]. The authors explored the changes due to breeding activity and the effect of the environment due to different rainfall distribution during grain filling. The GxE interaction was significant, also due to the large genotypic variability, especially in terms of earliness. A lower WE-AX and AX solubility were observed in the dryer crop season (in semolina and in wholemeal), in accordance with the relationship between rainfall and WE-AX already described in bread wheat before. These changes were also related to variations in some AXOS (both within mono or di-substituted) that influenced also the viscosity of the aqueous extracts. The authors reported also the environmental influence of MLG, with higher content in wholemeal in the dryer crop season, associated to a reduction of the ratio of G3:G4 GOSs. Similarly, Nocente et al. [83], on durum wheat (cv. Colosseo) grown under organic and conventional farming in different crop seasons (central Italy, University of Tuscia), confirmed the role of rainfall on AX content for pasta quality.

### 3.3. Bioactive Compounds and Antioxidant Capacity

The role of the environment on the accumulation of bioactive compounds has been investigated mostly in bread wheat, and most of the studies reported a strong influence of genotype in interaction with environment. This is reported in literature for common wheat, as outcome from HEALTHGRAIN wheat diversity screening for phenolic acids [103], alkylresorcinols [104], tocopherols and tocotrienols [105], phytosterol [106], and folate [107]; in particular precipitations during grain filling are generally negatively correlated to the content of many bioactive compounds [85].

As for durum wheat, very few studies are reported in relation to the imposition of drought or salt conditions, in particular under controlled conditions. In the study conducted by [63,87], the effects of pre-anthesis water-deficit stress was investigated on phenolic content of both leaf tissues and whole grain in different durum wheat genotypes. The authors reported that phenolic acid (PA) content could be associated with water-deficit stress tolerance, since the higher phenolic accumulation was associated to water stress only in some tolerant genotypes. Free and bound PA were characterized by a GxE interaction; in addition, the changes observed in leaf PA accumulation correlated with those observed in grain. The authors the stated that the impacts of pre-anthesis water stress on the phenolic concentrations are genotype dependent. In another study conducted at Cimmyt [61] on six durum wheat lines grown in a field trial moderate and severe drought stress were compared to optimal full drip irrigation in order to investigate for PA content and composition. Genotype, growth conditions, year, and their interactions had a highly significant effect on phenolic acid content and environmental conditions were the most impactful sources of variation. The mean increase in total PA with severe drought stress was marked mainly for ferulic acid, which represented up to 90% of PA composition. On the other hand, hydroxybenzoic and sinapic acids showed a decrease with water stress.

Conversely, most studies on this topic are not based on a direct imposition of stress, but deal with the evaluation of bioactive content/composition and antioxidant properties of durum wheat grain in relation to the impact of genotype, as well as growing environment, including cropping years and/or cultivation areas, and the interaction of these factors.

In the study of Fratianni et al. [62], the combined effect of genotype, water deficit, and sulphur fertilization on carotenoid and tocol contents was investigated in both durum wheat wholemeal and semolina. As for genotypic effect, wholemeal carotenoid and tocol levels significantly varied under irrigated field conditions among the different tested cultivars. Interestingly, water deficit was found to induce an improvement of lipophilic antioxidants in durum wheat grains. Indeed, under rainfed conditions, Simeto showed a significant increase in wholemeal (20%) and semolina (15%) carotenoids, as well as in wholemeal (10%) and semolina (15%) tocols, while water stress induced only a 15% increase in Ofanto semolina carotenoids. On the other hand, sulphur supplementation was unable to improve the lipophilic antioxidant response under water deficit, as it positively affected mainly Ofanto wholemeal carotenoids, as well as semolina tocols/tocotrienols, under both water regimes [62].

However, these studies can provide interesting information from comparison of cropping years with very different climatic conditions, as well as of environments with different pedoclimatic characteristics, that can be assimilated to water deficit conditions; that gives information about the adaptability and stability in performance of crops in different growing areas. In the studies of Graziano et al. [58,88]**,** the levels and antioxidant properties of phenolic acids were investigated in durum wheat whole grains by comparing the response of different Italian cultivars (Cappelli, Saragolla, Iride, Svevo) in two consecutive growing seasons**.** Interestingly, significant differences were found for soluble and insoluble-bound phenols between the two cultivation years, with higher values in the drier crop season, showing lower rainfall and higher temperatures during grain filling. The amounts of these polyphenols were also statistically different in the different investigated environments, with the highest amounts in the driest environment [88]. Another field study investigated the profile and content of phenolic acids as well as antioxidant capacity (AC) of durum wheat genotypes grown in three different locations representing different durum wheat cultivation areas in Italy [89]. Genotype, environment, crop year, and their interaction had highly significant effects on both AC levels and PAs content, confirming the higher PA concentration during the drier crop years. In the same Italian locations (Jesi and Foggia), Bellato et al. [90] investigated the total soluble phenolic content, 5-n-alkylresorcinol (AR) content and AC of 30 durum wheat cultivars grown in two successive growing seasons. Significant but low negative correlations were found between ARs and phenolic content and between total phenolic content and AC, but not between ARs and AC. Both environment and genotype showed highly significant effects on AR and total phenolic contents as well as on AC, with highly significant genotype x environment interactions. The highest AR concentrations were recorded in the dry environment (Foggia in 2010) showing the lowest amounts of rainfall during grain filling; conversely, high precipitation and water availability during grain filling appeared to increase the accumulation of soluble phenols. A significant influence of environment on the antioxidant properties was also found, but no relationship between AC and total precipitation. The content of ARs, yellow pigments, and total phenolic compounds, as well as AC, were also investigated in grains of cv. Colosseo derived from a field experiment conducted in two consecutive (2009–2010 and 2010–2011) growing seasons at Viterbo (central Italy) under both organic and conventional management practices by using conventional and reduced soil tillage system [83]. A significant effect of cropping year was observed for all investigated traits (except for total phenols), with higher values measured in the highly rainy growing season, thus showing the relevant impact of environmental and climate conditions on the nutraceutical properties of durum wheat grains, also in relation to cropping system and soil tillage.

Some studies investigated the changes in bioactive compound composition as affected by environmental factors in durum wheat grains in comparison with other cereals. AC and total polyphenolic and flavonoid contents were studied in grains from six modern and three old durum wheat varieties, in comparison with 17 modern and old soft wheat varieties, cultivated in the same site (Florence, central Italy) during two consecutive crop seasons (2006–2007 and 2007–2008) under an organic agricultural system [91]. Both total phenolic and flavonoid content, as well as AC, were found to significantly differ in the two years for both durum and soft modern and old wheat varieties. In particular, they resulted much higher in the crop season showing lower temperatures and higher rainfall in the 30 days before harvesting. Differences in PA content/composition and AC were also investigated in 39 wheat accessions belonging to different diploid (*Triticum monococcum*, einkorn), tetraploid (*Triticum turgidum* ssp. *dicoccum*, *turanicum* and *durum*), and hexaploid (*Triticum aestivum* ssp. *spelta and aestivum*) *Triticum* species, cropped for two years (2008 and 2011) in the same location (S. Angelo Lodigiano, northern Italy) [92]. A significant effect of the cropping year was recorded for the conjugated phenolic acids (except for *p*-hydroxybenzoic acid). In particular, a higher concentration (on average +15%) was found during the crop year (2011) showing minimal rainfall during the heading and maturing stages. Interestingly, the drought-stress induced increase was more pronounced among the *T. turgidum* than the *T. aestivum* and *T. monococcum* samples. On the contrary, the bound PAs generally did not change considerably from year to year. The effect of genotype, environment, and their interaction on total phenolic content of durum wheat whole grains was also investigated in comparison with soft wheat, oat, barley, and triticale **[93]**. To this aim, the more widely cultivated or more recent cultivars of each species (30, 25, 24, 12, and 10 for durum wheat, soft wheat, barley, triticale, and oat, respectively) were grown in two or three places selected among four Italian locations geographically located at North, Center, and South. The location factor showed a significant effect for durum wheat, as well as for other tested cereal species. Genotype and genotype x environment interaction were significant for durum wheat, as well as for other cereals with the exception of oat and barley, respectively. Environment resulted the main factor influencing the accumulation of phenolic compounds in durum wheat grains.

As for lipid composition in grain, a genotypic variability is known in terms of durum wheat fatty acids, with linoleic acid (18:2) representing the main fatty acid in grains [108]; indeed, recent lipidomic study confirmed that the progressive reduction in free fatty acids (FFA) during grain filling is associated to a predominant accumulation of l8:2, also as phospholipids and galactolipids since two weeks after anthesis [109]; in a further lipomic study on durum wheat obtained in Mediterranean environment (Italy), the authors found that lipid content and composition was stable across crop seasons [108].

As for the effect of salinity stress, in a study conducted in greenhouses under three irrigation water salinity levels (0.9, 6.0, and 12.0 dS m^−1^) wholemeal carotenoid content was also determined in 10 durum wheat genotypes of different origin and release date [35]. An improvement in grain carotenoid pigment on increasing salinity level was observed, consistently with a yield decrease. With regard to genotypic response to salt stress, a high variability was detected, with a significant effect of genotype–salinity interaction [35].

### 3.4. Micronutrients

Micronutrients malnutrition is often associated with diets rich in cereals [17]. Biofortification has been suggested as a sustainable solution to improve food security and nutritional quality, since wheat is a staple source of dietary energy for millions of people, including poor consumers, with Zn considered as primary target [110]. Micronutrients in grain are generally affected by a complexity of factors, including soil content, water availability, and genetic variability [19]. In general, water deficit reduces grain carbon (starch) accumulation and, consequently, increases mineral (ash) concentration [51].

In a study of Magallanes-López et al. [60] at Cimmyt, 46 durum wheat varieties have been analyzed in a field trial under full and reduced irrigation in order to evaluate the role of drought in micronutrient uptake in relation to genetic diversity. The authors reported a mean slight increase in Fe grain concentration under reduced irrigation with respect to full water supply, not observed in relation to Zn. The authors associated the response in Zn concentration to the limited variation observed in grain weight due to drought. Further, the authors observed a significant reduction in phytic acid with limited water supply; this was put in relation to Fe and Zn concentration, suggesting potential better micronutrient bioavailability for durum wheat under water stress condition. The same authors observed, in a smaller group of durum wheat genotypes, an increase in Fe under water stress [65].

In a further field experiment conducted in Italy under rainfed conditions, mineral content was investigated in relation to genetic variability and breeding activity, in 84 genotypes of different release date [16]. The field trials were conducted in three environments with different cumulated rainfall amounts (Foggia 2004/05 455 mm, Foggia 2005/06 548 mm, Fiorenzuola D’Arda 750 mm). Higher mineral concentration was generally observed in the dryer environments, in particular for Ca, Fe, and Mg, while Mg and Zn were influenced by soil content. Additionally, in the study reported by Galieni et al. [20], the higher Zn accumulation was observed in the dryer crop season, with a positive relationship with fertilization N rate. Limited information is available in relation to salt stress on durum wheat micronutrient uptake and grain concentration; however, one of the effect of high salinity level (in particular Na^+^), is in terms other ions imbalance that can lead to differential accumulation of minerals in wheat grain [54].

## 4. Conclusions

Durum wheat is a species well adapted to Mediterranean climate where hyperosmotic stress conditions may have an increasing frequency in the next years due to climatic changes.

Both water deficit and salinity may have a great impact on durum wheat grain quality. In particular, the hyperosmotic stresses have an impact on C and N metabolism during grain filling which may influence both content and composition of the main durum wheat quality markers. Protein content and composition have a strong influence on technological quality, both for pasta and bread-making. There is a general consensus on the increase of protein content under drought associated to a higher concentration of high molecular weight sulfur-poor prolamins, in particular ω-gliadin and HMW-GS; the higher protein content has also a positive impact on technological quality which is highly dependent on glutenin composition, especially B-type LMW-GS and HMW-GS 7+8/6+8. As for health quality, the possible higher accumulation of S-poor gliadins could lead to an upregulation of Tri a 19 allergen; on the basis of the recent scientific literature on durum wheat, also the accumulation of CD-epitope containing proteins seems to be influenced by environment and rainfall. The effect of hyperosmotic stress on dietary fiber is contrasting; however, the reduction of the water insoluble fractions of dietary fiber may enhance the positive benefit of durum wheat in Mediterranean diet, especially of wholemeal flours. Stressed durum wheat grains generally tend to accumulate bioactive compounds, in particular phenolic acids (ferulic ac.), alkylresorcinol, and carotenoids, although the interaction with the genotype is significant for these traits. Finally, as an indirect effect of reduced starch accumulation, a higher mineral content, especially of iron is reported for durum wheat, as a positive effect on human nutrition. Further investigation on the influence of hyperosmotic stress on durum wheat physiology and yield performance will be necessary with a multidisciplinary approach, in order to obtain high quality durum wheat adapted to the upcoming environmental limitations.

## Figures and Tables

**Table 1:** Effects of water deficit and salt stress on durum wheat grain composition for the main quality traits (protein, dietary fiber, micronutrients, bioactive compounds).

Plant Genotype	Growth Conditions	Water Deficit/Salinity Conditions (mm Rainfall + Irrigation/Salt Stress Level)	Traits Investigated	References
10 durum wheat genotypes	Field, irrigation	Irrigated optimal control (311 mm + 150 mm) vs. one mild drought stress rainfed (330 mm + 0 mm) and one severe drought stress rainfed (188 mm + 0 mm)	GPC, AA composition	[64]
2 durum wheat genotypes (Ofanto, Simeto)	Field, irrigation	Irrigated (I, ~ +45 mm) vs. not irrigated (NI) in three crop seasons, (~230 mm mean rainfall)	GPC, gliadin, glutenin, HMW-GS, HMW-GS/LMW-GS, UPP	[34]
6 durum wheat genotypes	Field, irrigation	One full irrigation (500 mm) vs. one moderate drought stress (300 mm) and one severe drought stress (180 mm)	GPC, HMW-GS, LMW-GS, ω-gliadin, γ-gliadin, α-gliadin	[55,65]
2 durum wheat genotypes (Ciccio, Svevo)	Growth chamber, irrigation	Well-watered (WW, 9522 mL) vs. water stress (WS, 7920 mL)	Gluten proteome, HMW-GS, LMW-GS, ω-gliadin, γ-gliadin, α-gliadin	[56,66]
1 durum wheat genotype (Iride)	Field, rainfed	Two crop seasons (507.2 mm in 2010/11, 314.6 mm in 2011/12)	GPC, gliadin, HMW-GS, LMW-GS, minerals	[20,67]
1 durum wheat genotype (Saragolla)	Field, rainfed	Two crop seasons (290.2 mm in 2015/16, 153.8 mm in 2016/17)	GPC, gliadin, HMW-GS, LMW-GS, total GS/glia, HMW-GS/LMW-GS	[68]
15 durum wheat genotypes (7 old, 8 modern)	Field, rainfed	Two crop seasons (293 mm in 2012/13, 310 mm in 2013/14—reproductive stages 54 mm in 2012/13, 153 mm in 2013/14)	GPC, glia/glut, HMW-GS, LMW-GS, ω-gliadin, γ-gliadin, α-gliadin, HMW-GS/LMW-GS, gluten index, UPP, AA composition, Tri a 19, TECP, IECP, G12	[57,69,70]
2 durum wheat genotypes (Cappelli, Saragolla)	Field, rainfed	Two crop seasons (in reproductive stages 181 mm in 2015/16, 55 mm in 2016/17)	GPC, gliadin fractions, HMW-GS, LMW-GS, HMW-GS/LMW-GS, glia/glut, AA composition, IP, TP, free, conjugated, bound and total phenolic acids	[58]
6 durum wheat genotypes	Field, rainfed	Four locations and two crop seasons in Italy (differences in rainfall in 8 environments)	GPC, HMW-GS, LMW-GS, gliadin, IP, TP	[71]
79 durum wheat genotypes	Field, rainfed	Two crop seasons (289 mm in 2015/2016 to 209 mm in 2016/2017)	GPC, HMW-GS, LMW-GS, gliadin, IP, TP	[72]
6 durum wheat genotypes	Field, rainfed	Two crop seasons (754 mm in 2003/04, 542 mm in 2004/05—in spring 210mm in 2003/04, 79 mm 2004/05)	GPC, gliadin/glutenin	[73]
16 durum wheat (12 old, 2 intermediate, 2 modern)	Field, rainfed	Two crop seasons (363 mm in 2015/16, 286 mm in 2016/17)	GPC, ω-gliadin, γ-gliadin, α-gliadin, glia/glut, HMW/LMW, S-rich/S-poor, UPP	[74]
1 durum wheat genotype (Creso)	Field, rainfed	Two sowing dates, four N levels (N0, N6, N12, N18), two crop seasons (250/159 mm in 2003, 530/304 mm in 2005—in spring 25/8 mm in 2003, 69/30 mm in 2005)	GPC, gliadins, glutenins, SPP, LPP, UPP	[75,76]
2 durum wheat genotypes	Greenhouse	Three salinity levels (0.9, 4.0, 8.0 dS/m)	GPC, gluten, SDS-sedimentation test,β-carotene	[77]
10 durum wheat genotypes	Greenhouse	Three salinity levels (0.9, 6.0, 12.0 dS/m)	GPC, SDS-sedimentation test, carotenoid	[35]
2 durum wheat genotypes (Neodur, Virgilio)	Open-top chamber	Saline water (+S, 8.3 dS/m) vs. tap water (-S)	GPC, gluten	[78]
1 bread wheat genotype	Pot	Six NaCl% levels (0%, 0.15%, 0.30%, 0.45%, 0.6%, 0.75%)	HMW-GS	[79]
5 genotypes	Field	Two sites in France, Auzeville (rainfed) and Melgueil (rainfed and irrigated, +102 mm by sprinkler)	Tot-AX, WE-AX, WE-AX/WU-AX, ferulic acid	[80]
19 durum wheat genotypes	Field	Two sites and two crop seasons (616 mm Jesi 2008/09, 702 mm Jesi 2009/10; 749 mm Foggia 2008/09, 440 mm Foggia 2009/10)	Tot-AX, WE-AX, A/X	[81]
15 durum wheat genotypes (7 old, 8 modern)	Field	Two crop seasons (293 mm in 2012/13, 310 mm in 2013/14—reproductive stages 54 mm in 2012/13, 153 mm in 2013/14)	In semolina and whole meal, Tot-AX, WE-AX, β-glucan, AXOS composition, GOS composition	[7,82]
1 durum wheat genotypes (Colosseo)	Field	Two crop seasons (2010 > 2011)	Tot-AX, alkylresorcinols, total phenols, AC	[83]
26 bread wheat genotypes	Field	6 environments with different rainfall amounts Hungary (~ 426 mm in 2004/05 and 2005/06, ~315 mm in 2006/07) Poland (~ 426 mm in 2006/07) France (~315 mm in 2006/07) UK (~689 mm in 2006/07)	In flour and bran. Total DF, total NSP, WENSP, Tot-AX, WE-AX, lignin, β-glucan	[84,85]
3 bread wheat genotypes14 bread wheat/Aegilops. spp. lines	Growth chamber	Control (SWC 30-35% vs. drought stress (SWC 10–15%)	GPC, glutenin/gliadin, UPP, Tot-AX, WE-AX, β-glucan, AXOS composition, GOS composition	[59,86]
46 durum wheat genotypes	Field	One full irrigation (500 mm) vs. one moderate drought stress (300 mm), mDS vs. opt, −40% water	Fe, Zn, phytic acid	[60]
84 durum wheat genotypes	Field	Two crop seasons (455 mm in 2004/05, 548mm in 2005/06)	Minerals (P, Ca, Cu, Fe, K, Mg, Mn, Na, Zn), phytic acid	[16]
6 durum wheat genotypes	Field	One full irrigation (500 mm) vs. one moderate drought stress (300 mm)	Total phenolic acids, p-Hydroxybenzoic, Syringic, Vanillic, p-Coumaric, Ferulic, Sinapic	[61]
4 durum wheat genotypes	Field, irrigation	Two crop seasons (grain filling 67 mm first year, 24 mm second year), irrigated vs. rainfed	Carotenoids, tocopherols, and tocotrienols in whole meal and semolina	[62]
8 durum wheat genotypes	Glasshouse	Control conditions (12% SWC, from germination to maturity) vs. water-deficit stress (6% SWC from booting to maturity)	Free, conjugated, bound, and total phenolic acids	[63,87]
2 durum wheat genotypes (Iride, Svevo)	Field	Four locations and two crop seasons in Italy (differences in rainfall amount and distribution)	Free, conjugated, bound, and total phenolic acids, AC	[88]
30 Italian durum wheat genotypes	Field	Three locations and two crop seasons in Italy (differences in rainfall amount and distribution)	Free, conjugated, bound, and total phenolic acids, AC	[89]
30 Italian durum wheat genotypes	Field	Two sites and two crop seasons (616 mm Jesi 2008/09, 702 mm Jesi 2009/10; 749 mm Foggia 2008/09, 440 mm Foggia 2009/10)	Total soluble phenols, alkylresorcinol	[90]
2 old and 6 modern durum wheat genotypes	Field	Two crop seasons (30 days before harvest 80 mm in 2007, 29.6 mm in 2008).	Soluble phenols, AC	[91]
13 accessions of tetraploid wheats (including durum wheat and emmer)	Field	Two crop seasons	Conjugated and bound phenols, AC	[92]
30 durum wheat genotypes	Field	One crop season and three sites in Italy, north (Fiorenzuola d’Arda) central (Larino and Matrice), south (Foggia)	Total phenols	[93]

GPC = grain protein content; AA = amino acid; HMW-GS = high molecular weight glutenin subunits; UPP = unextractable polymeric proteins; GI = gluten index; AlvW = alveographic dough strength; AlvP = alveographic dough tenacity; AlvL = alveographic dough extensibility; AlvP/L = alveographic dough tenacity to extensibility ratio; glia/glut = gliadin to glutenin ratio; Tri a 19 = ω5-gliadin wheat allergen; IECP = immunogenic epitope-containing peptides; TECP = toxic epitope-containing peptides; G12 = monoclonal antibody specific to a sequence included in the celiac disease-related 33-mer epitope; IP = immunogenic peptides; TP = toxic peptides; S-rich/S-poor = ratio between Sulphur-poor to Sulphur-rich storage proteins; SPP = small polymeric proteins; LPP = large polymeric proteins; Tot-AX = total arabinoxylan; WE-AX = water extractable arabinoxylan; WU-AX = water unextractable arabinoxylan; AXOS = arabinoxylan oligo-saccharides; GOS = gluco oligo-saccharides; DF = dietary fiber; NSP = non-starch polysaccharides; WENSP = water extractable non-starch polysaccharides; PA = phenolic acid; AC = antioxidant capacity; SWC = volumetric soil water content.
